# Polycystic Ovary Syndrome and Metabolic Syndrome: Clinical and Laboratory Findings and Non-Alcoholic Fatty Liver Disease Assessed by Elastography

**DOI:** 10.1055/s-0041-1741032

**Published:** 2022-05-16

**Authors:** Claruza Braga Holanda Lavor, Antonio Brazil Viana Júnior, Francisco das Chagas Medeiros

**Affiliations:** 1 Department of Gynecology, Maternidade Escola Assis Chateaubriand, Universidade Federal do Ceará, Fortaleza, CE, Brazil; 2 Research Management Department, Hospital Universitário Walter Cantídio, Universidade Federal do Ceará, Fortaleza, CE, Brazil; 3 Department of Gynecology, Universidade Federal do Ceará, Fortaleza, CE, Brazil

**Keywords:** elastography, ultrasonography, polycystic ovaries, metabolic syndrome, obesity, elastografia, ultrassonografia, ovários policísticos, síndrome metabólica, obesidade

## Abstract

**Objective** To evaluate the association between polycystic ovary syndrome (PCOS) and metabolic syndrome (MetS), adding liver assessment through elastography and ultrasound, for correlation with non-alcoholic fatty liver disease (NAFLD). Metabolic syndrome occurs in ∼ 43% of women with PCOS, and NAFLD is the hepatic expression of MetS.

**Methods** One hundred women, 50 with PCOS and 50 controls, matched by age (18–35 years) and body mass index (BMI) were included, restricted to patients with overweight and obesity grade 1, at the Assis Chateaubrian Maternity School, Universidade Federal do Ceará, Brazil. For the diagnosis of PCOS, we adopted the Rotterdam criteria, and for the diagnosis of MetS, the criteria of the National Cholesterol Education Program (NCEP/ATP III). Hepatic elastography and ultrasound were performed to assess liver stiffness and echotexture, respectively.

**Results** The average ages were 29.1 (±5.3) and 30.54 (±4.39) years, for the PCOS and the control group, respectively. Patients with PCOS had a risk 4 times higher of having MetS, odds ratio (95% confidence interval) = 4.14, than those in the control group. Women with PCOS had higher average of abdominal circumference (100.9 ± 9.08 cm *vs* 94.96 ± 6.99 cm) and triglycerides (162 ± 54.63 mg/dL *vs* 137.54 ± 36.91 mg/dL) and lower average of HDL cholesterol (45.66 ± 6.88 mg/dL *vs* 49.78 ± 7.05 mg/dL), with statistically significant difference. Hepatic steatosis was observed on ultrasound in women with PCOS; however, with no statistically significant difference. There was no change to NAFLD at elastography in any group.

**Conclusion** Women with PCOS had 4-fold higher frequency of MetS and more hepatic steatosis, with no statistically significant difference. There was no change in liver stiffness between the groups at elastography. The results can be extended only to populations of overweight and obesity grade 1, with PCOS or not. They cannot be generalized to other untested groups.

## Resumo

**Objetivo** Avaliar a associação entre a síndrome do ovário policístico (SOP) e a síndrome metabólica (SM), agregando avaliação do fígado por elastografia e ultrassonografia, para correlação com doença hepática gordurosa não alcoólica (DHGNA). A SM ocorre em cerca de 43% das mulheres com SOP, e DHGNA é a expressão hepática da SM.

**Métodos** Foram incluídas 100 mulheres, pareadas por idade (18–35 anos) e índice de massa corporal (IMC), 50 com SOP e 50 controles com sobrepeso e obesidade grau I, na Maternidade-Escola Assis Chateaubriand, Brasil. Para o diagnóstico de SOP, adotamos os critérios de Rotterdam e, para o diagnóstico de SM, os critérios do *National Cholesterol Education Program* (NCEP/ATP III). Elastografia hepática e ultrassonografia foram realizadas para avaliar a rigidez e a ecotextura do fígado, respectivamente.

**Resultados** As médias de idade foram de 29,1 (±5,3) e 30,54 (±4,39) anos para os grupos SOP e controle, respectivamente. Pacientes com SOP apresentaram risco 4 vezes maior de SM do que aquelas no grupo controle [[razão de chances (intervalo de confiança de 95%) = 4,14]. Mulheres com SOP tiveram maior média de circunferência abdominal (100,9 ± 9,08cm *vs* 94,96 ± 6,99 cm) e triglicérides (162 ± 54,63 mg/dL *vs* 137,54 ± 36,91 mg/dL) e menor média de colesterol HDL (45,66 ± 6,88 mg/dL *vs* 49,78 ± 7,05 mg/dL), com diferença estatisticamente significativa. Esteatose hepática foi observada em ultrassonografias de mulheres com SOP, porém sem diferença estatisticamente significativa. Não houve mudança para DHGNA na elastografia em nenhum dos grupos.

**Conclusão** Mulheres com SOP tiveram frequência quatro vezes maior de SM e mais esteatose hepática, sem diferença estatisticamente significativa. Não houve mudança na rigidez do fígado entre os grupos na elastografia. Os resultados podem ser estendidos apenas a populações com sobrepeso e obesidade grau 1, com SOP ou não. Eles não podem ser generalizados para outros grupos não testados.

## Introduction

Polycystic ovary syndrome (PCOS) is the most common endocrine disorder among women in menacme, associated with both reproductive abnormalities and hyperandrogenic and metabolic changes. Its etiology remains largely unknown, but it is considered a complex disorder, with associated genetic and environmental factors. 1 2 The diagnostic criterion most used clinically for the diagnosis of PCOS is the Rotterdam, in which it is necessary to have two of the following three criteria: 1) clinical or laboratory hyperandrogenism; 2) oligo/anovulation; 3) micropolicystic ovaries on ultrasound. Depending on the diagnostic criteria used, PCOS affects between 4 and 19% of women of reproductive age. 3

The association of risk factors, including obesity, predisposes to greater morbidity, and mortality due to systemic metabolic changes. Metabolic syndrome (MetS) includes abdominal obesity, altered carbohydrate metabolism, dyslipidemia, endothelial dysfunction, and arterial hypertension, being associated with an increased risk of developing cardiovascular diseases. 4 5

The definition of MetS 6 proposed by the National Cholesterol Education Program (NCEP) is the most recommended one for use in the clinic, due to its simplicity and practicality. According to this definition, women who have three or more of the following criteria are classified as having MetS: waist circumference (WC) ≥ 88 cm, increased triglyceride (TGC) levels (> 150 mg/dL), reduced high-density cholesterol (HDL-C < 50 mg / dL), systemic arterial hypertension, and hyperglycemia (blood glucose ≥ 110 mg / dL). 6 7

Similar to what occurs in PCOS and MetS, insulin resistance (IR) also arises from the pathogenesis of individuals with non-alcoholic fatty liver disease (NAFLD), the most common form of liver disease today. Thus, insulin-resistant patients are at greater risk of developing NAFLD due to its evolutionary potential, especially in individuals with MetS. Interventions for early and therapeutic diagnosis are of great value for these patients. 8 The high prevalence of obesity and IR in patients with PCOS, as well as hyperandrogenism, are the main drivers of the increased risk for NAFLD in this population. 9

Given the above, there was a need to assess the association between PCOS and MetS, adding measurement of stiffness and assessment of liver echotexture through elastography and ultrasound, respectively, for correlation with NAFLD, comparing women with and without PCOS.

## Methods

A study was performed with women seen at an outpatient clinic specialized in gynecology-endocrine at Maternidade-Escola Assis Chateaubriand, UFC, Brazil, between April, 2019 and October, 2019. The Hospital's research ethics committee previously approved the clinical protocol, and all patients signed the consent form. It included a total of 100 women: 50 with PCOS and 50 without PCOS. They were matched for age (18–35 years) and body mass index (BMI), and the study was restricted to patients with overweight and obesity grade 1, not being able to generalize to other untested group. Patientes were randomly recruited according to the inclusion and exclusion criteria.

The PCOS group included women diagnosed with PCOS (based on the Rotterdam criteria), not using hormonal contraception for at least 3 months, with or without criteria for metabolic syndrome. The non-PCOS group included women without a diagnosis of PCOS, not using hormonal contraception for at least 3 months, with regular menstrual cycles (28–32 days), transvaginal ultrasound with normal ovarian morphology (< 12 follicles in each ovary, or ovarian volume <10 cm ^3^ ), and without clinical signs of hyperandrogenism.

Those women who had the following characteristics were excluded: pregnant women; women with a history of chronic alcoholism; women with chronic liver disease (hepatitis-B virus [HBV] or hepatitis-C virus [HCV] positive; alteration of glutamic-oxalacetic transaminase [TGO] and glutamic pyruvic transaminase [TGP]); women who underwent liver surgical procedure; women who used risk-factors drugs for NAFLD (corticosteroids, tamoxifen), and drugs that could interfere with blood glucose levels (metformin), or for the treatment of dyslipidemia (statins).

Weight groups were defined by the BMI, according to the World Health Organization (WHO): BMI overweight (25–29.9 kg / m ^2^ ) and BMI obesity grade 1 (30–34.9 kg/m ^2^ ). 10 The height and weight of the patients were obtained, and the BMI was calculated according to the formula weight (Kg)/height (m ^2^ ). Blood pressure was measured by the average of the results of 2 pressure measurements in mmHg, in a sitting position, each after at least 10 minutes of rest. The presence of signs of hyperandrogenism (mainly the presence of hirsutism—face, breasts, abdomen, thigh roots, buttocks, back) and acanthosis nigricans (neck, armpits) was also evaluated. Blood samples were requested to be collected in the morning, after 12 hours of overnight fasting.

Regarding laboratory tests, patients under investigation for PCOS underwent tests to exclude other endocrinopathies (hyperprolactinemia, hypothyroidism, and congenital adrenal hyperplasia) such as prolactin, thyroid-stimulating hormone (TSH) and 17 OH progesterone. In addition to these, follicle-stimulating hormone (FSH), luteinizing hormone (LH), estradiol, total testosterone, and sex hormone binding blobulin (SHBG) were requested to assess the hormonal profile related to PCOS-related anovulation and hyperandrogenism. The homeostatic model assessment for insulin resistance (HOMA-IR) method was used to determine IR (values above 2.15), calculated according to the formula fasting glucose (mg/dL) x fasting insulin (μUI/mL)/22.5. 11

The diagnosis of PCOS was defined in the interview by the presence of at least two Rotterdam criteria (anovulation; hyperandrogenism; polycystic ovarian morphology at transvaginal ultrasound), excluding other endocrinopathies. Anovulation was defined by the presence of oligo/amenorrhea (menstrual interval of more than 35 days), and hyperandrogenism by the clinical presentation of hirsutism. The ovarian morphology at ultrasound had as standard the presence of 12 or more follicles, measuring 2 to 9 mm each, in at least one ovary, and/or ovarian volume > 10 cm ^3^ . 12 The definition of MetS chosen for this study was the one proposed by the NCEP - ATP III, 2005, which is the most recommended for clinical use. 13

## Hepatic Elastography

The elastography was performed on a Philips Affiniti 70 Ultrasound Machine, with acoustic radiation force impulse (ARFI) elastography software (). The method quantifies the mechanical properties of the liver using a high-intensity acoustic pulse to assess tissue elasticity, thus evaluating its mechanical response. The speed of the shear wave, recorded in m/s, is proportional to the degree of liver stiffness. 14

The volunteers had a 6-hour fasting preparation. The examination was performed with the patient in the supine position, with the right upper limb abducted. The region of interest (ROI) is a 10 × 5-mm rectangle, positioned inside the hepatic parenchyma, specifically in segment V of the right lobe, free of vessels, and under visual control by mode B, whose transducer can be moved freely. The measurements were made using an intercostal approach, with an acquisition sample 2 to 3 cm below the liver capsule. Ten measurements were obtained from independent images, in the same location, with the patient performing mild expiratory apnea to capture the speed measurements. The variability, which is the interquartile interval (IQR) between the measurements, should be < 30%, and it is the most important criterion for the reliability of the result, since it assesses the dissiparity of the values. 9

The results obtained from the ARFI were described by the median of the values of the wave propagation speed and by the IQR, according to the results presented by Friedrich-Rust et al. 12 : F0 (without fibrosis); F1 (initial fibrosis); F2 (intermediate fibrosis); F3 (advanced fibrosis), and F4 (fibrosis or cirrhosis nodules), as shown in Table 1 .The presence of speed values < 1.34 m/s excludes the presence of clinically significant fibrosis, with a high degree of certainty, as long as the patient does not present any other clinical and laboratory evidence of liver disease. 15 16

**Table 1:** Correlated acoustic radiation force impulse velocity measurements for liver fibrosis

ARFI	Cut-off (cut-off point of speed)
F0/F1	< 1.34m/s
F ≥ 2:	≥ 1.34m/s
F ≥ 3:	≥ 1.55 m/s
F = 4:	≥ 1.80 m/s

Abbreviation: ARFI, acoustic radiation force impulse.

## Ultrasonographic Diagnosis of NAFLD

All patients underwent liver ultrasound with a Philips Affiniti 70 3.5 MHz convex transducer, (Philips Healthcare, Cambridge, MA, USA). For the screening of hepatic steatosis, the echogenicity of the liver parenchyma was evaluated and compared with the texture of the spleen echo. When isoechogenic, the liver parenchyma was considered normal, that is, without evidence of steatosis. The presence of hyperechoic liver parenchyma was considered a characteristic of hepatic steatosis. 17 Steatosis was classified into:

- Level I (mild) when there is a diffuse increase in echogenicity in the liver parenchyma, but it allows a good view of the vessel walls;
- Level II (moderate) when there is a diffuse increase in echogenicity of the hepatic parenchyma, making it difficult to visualize the vessels and diaphragm;
- Level III (severe) when there is a significant increase in echogenicity with fine echoes and intense posterior attenuation, preventing the visualization of the vascular walls and diaphragm.

### Statistical Analysis

The data were entered into the RedCap platform, and the diagnostic agreement analysis was performed. The statistical analysis was performed on the IBM SPSS Statistics for Windows, version 22.0 (IBM Corp., Armonk, NY, USA), and the R 3.3.1 software. In numerical variables, the data were presented as mean, median, and standard deviation. In the categorical variables, the data were exposed to frequency and prevalence rate (percentage) to investigate associations between PCOS and MetS. The Student *t* -test and the Mann-Whitney U test were used to analyze the characteristics of the groups, conditioned to the data adherence to the Gaussian distribution. To investigate the association between categorical variables, the Pearson chi-squared test, and the Fisher exact test were used. A significance level of 5% was adopted ( *p* -values < 0.05 were considered statistically significant). Statistical analyses were performed using the statistical software R 3.3.1 and Jamovi 0.9.2.8.

## Results

A total of 100 women with the same BMI profile (overweight or grade 1 obesity) were included in this study, 50 with a diagnosis of PCOS and 50 controls (non-PCOS), and underwent metabolic analysis. The women in the PCOS group had oligoamenorrhea (100%) and polycystic ovarian morphology on ultrasound (100%). The women in the control group had a regular menstrual cycle (between 28–32 days) and normal ovaries on ultrasound.

The sample consisted of women aged between 18 and 35 years, with an average of 29.1 (±5.3) years for the PCOS group, and 30.54 (±4.39) years for the control group. The weighting profile showed an average BMI of 31.54 (±2.3) Kg/m ^2^ for the PCOS group, and 30.78 (±1.86) Kg/m ^2^ for the control group. There was no statistical difference between age and BMI ( *p* = 0.072 and *p* = 0.083, respectively). No patient had comorbidities or a history of alcoholism, and liver disease was also excluded after laboratory evaluation in cases and controls.

Analysis of criteria for Metabolic syndrome: For systolic blood pressure (SBP) and diastolic blood pressure (DBP), there was no difference between the groups ( *p* = 0.66 and *p* = 0.537, respectively). As for the abdominal circumference, a statistically significant difference was detected between the PCOS and control groups ( *p* = 0.001), in which the PCOS group presented a higher average of abdominal circumference (100.9 ± 9.08 cm vs 94.96 ± 6.99 cm). A difference was also observed in the analysis of fasting blood glucose measurements, with *p* = 0.011, in which the PCOS group had a higher fasting blood glucose, with an average of 92.6 ± 12.06 mg / dL vs 87.0 ± 10.27 mg / dL. The measurements of triglycerides and HDL cholesterol were evaluated and showed a statistically significant difference ( *p* = 0.010 and *p* = 0.004, respectively), as observed in Table 2 .

**Table 2:** Variations in the measures of the criteria for metabolic syndrome

Variables	Control (non-PCOS)			PCOS			*p*
	Average	Standard deviation	Median	Average	Standard deviation	Median	
SBP (mmHg)	115.00	8.39	120.00	116.30	10.82	120.00	0.660 ^b^
DBP (mmHg)	74.00	7.82	75.00	75.40	9.52	80.00	0.537 ^b^
Abdominal circumference (cm)	94.96	6.99	93.00	100.90	9.08	99.00	0.001 ^b^
Fasting blood glucose (mg/dL)	87.00	10.27	85.50	92.60	12.06	91.00	0.011 ^b^
Triglycerides (mg/dL)	137.54	36.91	130.00	162.20	54.63	159.50	0.010 ^a^
HDL cholesterol (mg/dL)	49.78	7.05	51.00	45.66	6.88	45.50	0.004 ^a^

Abbreviations: HDL, High-density lipoprotein cholesterol; PAD, Diastolic blood pressure; PAS, Systolic blood pressure.

(a) Student *t* -test. (b) Mann-Whitney test.

There was a higher prevalence of all components of MetS in the PCOS group, with emphasis on changes in HDL-cholesterol, waist circumference, and triglycerides ( Table 3 ).

**Table 3:** Prevalence of metabolic syndrome components between groups

MetS component	Prevalence% (n)		
	Control ( *n* = 50)	PCOS ( *n* = 50)	Total ( *n* = 100)	p
HDLC < 50 mg/dL	19 (38.0%)	39 (78.0%)	58 (58.0%)	< 0.001 ^c^
AC ≥ 88 cm	41 (82.0%)	50 (100.0%)	91 (91.0%)	0.003 ^c^
TGC ≥ 150 mg/dL	19 (38.0%)	30 (60.0%)	49 (49.0%)	0.028 ^c^
GLIC ≥ 110 mg/dL	2 (4.0%)	4 (8.0%)	6 (6.0%)	0.678 ^d^
BP ≥ 130 × 85 mm Hg	2 (4.0%)	7 (14.0%)	9 (9.0%)	0.160 ^d^

Abbreviations: AC, abdominal circumference; BP, blood pressure; TGC, triglycerides; GLIC, fasting blood glucose; HDL-C, high-density lipoprotein cholesterol; PCOS, polycystic ovary syndrome.

Values expressed in n%.

(c) Pearson chi-squared test. (d) Fisher test

Insulin resistance was assessed by laboratory analysis of fasting blood glucose and insulin levels. Patients in the PCOS group had more IR by the insulin-HOMA index (18% *vs* 0%), with a statistically significant difference ( *p* = 0.003). The patients were evaluated clinically on physical examination by the presence of acanthosis nigrican (54% *vs* 6%; *p* < 0.001). Table 4 shows, in addition to data on IR, the percentage of hyperandrogenism, in which clinical hyperandrogenism, represented by hirsutism, was significantly higher in the PCOS group (52% *vs* 0%; *p* < 0.001).

**Table 4:** Evaluation of hyperandrogenism and insulin resistance

Variables	Group		*p*
	Control ( *n* = 50)	PCOS ( *n* = 50)	
Hirsutism	0 (0.0%)	26 (52.0%)	< 0.001 ^c^
Total altered testosterone	0 (0.0%)	3 (6.0%)	0.242 ^d^
Acanthosis nigricans	3 (6.0%)	27 (54.0%)	< 0.001 ^c^
Insulin - HOMA-IR changed	0 (0.0%)	9 (18.0%)	0.003 ^d^

Abbreviation: HOMA-IR, homeostatic evaluation model-insulin resistance

Data exposed in n (%).

(c) Pearson chi-squared test. (d) Fisher exact test.

It was shown that overweight or obesity-grade 1 patients diagnosed with PCOS have a risk 4 times higher of presenting MetS, odds ratio (95% confidence interval) = 4.14 (1.79–9.57) than overweight or obesity-grade 1 patients without PCOS, as shown in Table 5 .

**Table 5:** Risk of metabolic syndrome

Group	With MetS	Without MetS	*p*
PCOS ( *n* = 50)	32 (68.1%)	18 (34.0%)	0.001 ^c^
Control ( *n* = 50)	15 (31.9%)	35 (66.0%)	

Abbreviations: MetS, metabolic syndrome; PCOS, polycystic ovary syndrome.

OR (95% CI) = 4.14 (1.79 - 9.57);

(c) Pearson chi-squared test

Hepatic steatosis was observed on ultrasonography in the PCOS group vs control group (mild steatosis: 26% cases vs 18% controls; moderate steatosis: 10% cases vs 6% controls; accentuated steatosis: 4% cases vs 0% controls), with no statistically significant difference ( *p* = 0.184), as shown in Table 6 .

**Table 6:** Prevalence of liver changes in abdominal ultrasound

Variables	Group		*p*
	Control ( *n* = 50)	PCOS ( *n* = 50)	
Liver alteration			0.184 ^d^
Mild hepatic steatosis	9 (18.0%)	13 (26.0%)	
Moderate hepatic steatosis	3 (6.0%)	5 (10.0%)	
Marked hepatic steatosis	0 (0.0%)	2 (4.0%)	
Without changes	38 (76.0%)	30 (60.0%)	

Data exposed in n (%). (d) Fisher exact test

In the sample of patients from the PCOS group, the Fisher exact test detected a significant association between IR (HOMA-IR > 2.15) and NAFLD, *p* < 0.001 and Cramer V = 0.57, in which all women with IR had NAFLD, as shown in Table 7 .

**Table 7:** Association between insulin resistance and non-alcoholic fatty liver disease in patients with polycystic ovary syndrome

PCOS group ( *n* = 50)	Insulin resistance	
NAFLD	Without IR	With IR
With NAFLD	11 (27 %)	9 (100 %)
Without NAFLD	30 (73 %)	0 (0 %)

Abbreviations: IR, insulin resistance, NAFLD, non-alcoholic fatty liver disease, PCOS, polycystic ovary syndrome.

Fisher Exact test - *p* < 0.001 e Cramer's V = 0.57

The results of the ARFI elastography of the participants were expressed by the speed of the shear wave in the evaluation of the stages of hepatic fibrosis. There was no change to the NAFLD at elastography in any group, with the mean velocities being equal in the the PCOS and control groups (1.12 ± 0.13 m/s *vs* 1.12 ± 0.09 m/s, respectively; *p* = 0.664), according to Table 8 . To interpret the results, the speed value was correlated with the degree of fibrosis of the classification of Friedrich-Rust et al. 12 All women were at stage F0/F1 (speed < 1.34 m/s—without fibrosis or initial fibrosis).

**Table 8:** Speed and dissiparity values in elastography in women with metabolic syndrome

Variables	Group						
	Non-PCOS + MetS ( *n* = 15)			PCOS + MetS ( *n* = 32)			
	Average	Dp	Median	Average	Dp	Median	*p*
Speed (m/s)	1.12	0.09	1.12	1.12	0.13	1.13	0.664 ^b^
IQR	10.32	5.12	9.00	11.60	6.10	10.00	0.299 ^b^

Abbreviations: IQR, interquartile range; MetS, metabolic syndrome; PCOS, polycystic ovary syndrome.

Speed measurements in m/s. IQR measurements in percentage (%). (b) Mann-Whitney test

## Discussion

Metabolic syndrome is characterized by three main abnormalities: hyperglycemia, dyslipidemia, and obesity, which directly contribute to a proinflammatory state, predisposing to the development of DM2 and atherosclerotic cardiovascular disease. Hyperinsulinemia and IR are metabolic changes present in PCOS and MetS, inducing unfavorable changes in lipid metabolism and an increase in androgen production by ovarian teak cells. The excess of androgens in PCOS promotes dyslipidemia and abdominal adiposity, which contributes to the development of PCOS and MetS. This leads to a vicious circle of hyperinsulinemia, hyperandrogenism, central obesity, and metabolic abnormalities. 18

The present study, with volunteers aged 18 to 35 years, and BMI ranging from overweight to grade-1 obesity, shows a prevalence of MetS of 68% in women with PCOS, showing 4 times higher risk when compared with women without the diagnosis of PCOS. In comparison with a study by Dargham et al., 16 which involved women in the same age group and BMI, and the same diagnostic criteria for PCOS (Rotterdam criterion) and MetS (NCEP-ATP III), it was observed a prevalence of 58% for MetS. Women with PCOS also had more IR, higher BMI, higher waist circumference, and lower levels of HDL cholesterol, similar to the findings of this study. There was no difference between LDL cholesterol and blood glucose levels.

Echiburú et al. 17 also showed that women with PCOS had more MetS (5x higher) in menacme, at the expense of abdominal adiposity, IR, and increased cholesterol. Ferns and Ghayour-Mobarhan 5 performed a cohort study with several groups of women of different age groups in the region of Iran, using the Rotterdam criteria for the diagnosis of PCOS. The prevalence of MetS in PCOS was 28.8%, and they were considered a high-risk population for MetS. Their results for the MetS criteria were similar to those of this research, such as 72% with abdominal circumference ≥ 88 cm; 6% with blood glucose ≥ 110 mg/dL; 47% with triglyceride > 150 mg/dL; 86% with HDL cholesterol < 50 mg/dL.

In this sense, Melo et al. 18 concluded that, regardless of BMI, women with PCOS had more MetS, and the main defining criterion for MetS was HDL cholesterol < 50 mg / dL, which is a criterion also observed in this study. Alves et al. 19 added central obesity and hyperandrogenism as important factors for dyslipidemia and other metabolic disorders, negatively contributing to the long-term health of women with PCOS.

Taranto et al. 20 evaluated the association between NAFLD and PCOS demonstrating a high prevalence of steatosis in patients with PCOS (77%) when compared with patients with similar BMI but without PCOS (55%). Central adiposity (mean 103 cm) and serum triglyceride levels (mean 134 md/dL), two components of the MetS, were identified as important factors associated with steatosis, similar to the findings of this study. Insulin resistance was also associated with hepatic steatosis, which confirms its importance of IR in the pathophysiology of both conditions.

Although this study was not designed to clarify the causal relationship between PCOS, steatosis, and liver fibrosis, some hypotheses can be presented in light of the evidence in the literature. Non-alcoholic fatty liver syndrome and PCOS recognize the same dysmetabolic pathogenic background, that is, obesity and IR. In this scenario, hyperandrogenism related to PCOS can contribute to liver disease, promoting systemic inflammation, leading to decreased insulin sensitivity and hepatic fibrogenesis. On the other hand, NAFLD could further implement this vicious circle, contributing to IR, a key element in the pathogenesis of PCOS. 21

From a clinical point of view, it is suggested that patients with PCOS should be evaluated for the presence of hepatic steatosis, especially those with reduced insulin sensitivity and/or hyperandrogenism. In our study, no patient had elastography suggestive of moderate or severe fibrosis, probably due to our population being young and without diabetes, and not fitting in the obesity grade 2 or 3 category, being just overweight and obsesity grade 1. However, it is plausible that the persistence of IR and hyperandrogenism over time may lead to the progression of liver fibrosis and, consistently, these patients should be followed up to correct the risk factors mentioned above. 9

The present study included a population of patients with PCOS and overweight/obesity grade 1, which may differ in terms of metabolic characteristics and severity of liver disease from most cases of PCOS in the general population. The study can be extended only to populations of overweight and obese grade 1, with PCOS or not. They cannot be generalized to other untested groups.

The main limitation of this study lies in its cross-sectional nature, making it impossible to determine the temporal relationship between PCOS, IR, hyperandrogenism, and steatosis/liver injury. Another methodological issue is the use of non-invasive methods to detect steatosis and liver fibrosis, rather than liver biopsy. However, elastography is currently validated for the non-invasive assessment of NAFLD, while the liver biopsy is invasive and with potentially life-threatening complications, and it cannot be widely proposed in young patients with PCOS and healthy control patients.

Finally, our study included a population of PCOS patients, followed up at a tertiary referral center, which may differ in terms of metabolic characteristics and severity of liver disease from most PCOS cases in the general population. In these women with PCOS and controls, it was observed that PCOS quadrupled the risk of MetS, and its main determinants were obesity, IR, and hyperandrogenism. The data available in a recent meta-analysis by Ramezani-Binabaj et al. 22 observed that the prevalence of hepatic steatosis, assessed by US or magnetic resonance imaging, is higher in patients with PCOS compared with controls. We, then, observed that in patients with PCOS, the risk of steatosis was higher compared with controls matched for age and BMI; however, there was no statistical difference.

This study also showed that PCOS can be an independent risk factor for steatosis, and that IR and hyperandrogenism are the main factors of liver damage in PCOS. Published studies on the association between PCOS and NAFLD are still very scarce and recent, and they assess populations with lifestyles and genetic backgrounds different from those of the Brazilian population. It is observed that patients with PCOS have a higher incidence of MetS, hyperinsulinemia, overweight, increased abdominal circumference, and already begin to present changes in lipoproteins. Insulin resistance is a common pathogenic mechanism of these two entities and, therefore, it is expected that these women, if monitored and treated, may develop MetS and even NAFLD in the future. 23

Since MetS is commonly related to progression of liver disease, patients with PCOS presenting central adiposity and increased triglyceride levels should be screened for NAFLD. The doctor, especially the gynecologist, is expected to observe not only the aesthetic and reproductive effects but especially the metabolic and hepatic consequences of PCOS. Further studies to answer whether it is valid to monitor all patients with PCOS for NAFLD are needed for future referrals. 24 25 26 27

## Conclusion

There was a 4-fold higher frequency of MetS in women with PCOS. Abdominal obesity, dyslipidemia, and IR were the factors most related to metabolic risk. Concerning the assessment of NAFLD, women with PCOS had more hepatic steatosis, but without a statistically significant difference. There was no change between groups in hepatic stiffness at elastography. The results can be extended only to the overweight and obese grade-1 populations, with PCOS or not.
